# Prevalence and Genetic Diversity of Atypical Porcine Pestivirus (APPV) Detected in South Korean Wild Boars

**DOI:** 10.3390/v12060680

**Published:** 2020-06-24

**Authors:** SeEun Choe, Gyu-Nam Park, Ra Mi Cha, Bang-Hun Hyun, Bong-Kyun Park, Dong-Jun An

**Affiliations:** 1 Virus Disease Division, Animal and Plant Quarantine Agency, Gimchen, Gyeongbuk-do 39660, Korea; ivvi59@korea.kr (S.C.); changep0418@gmail.com (G.-N.P.); rami.cha01@korea.kr (R.M.C.); hyunbh@korea.kr (B.-H.H.); parkx026@korea.kr (B.-K.P.); 2 College of Veterinary Medicine, Seoul University, Gwanak-ro, Gwanak-gu, Seoul 08826, Korea

**Keywords:** APPV, wild boar, ML tree, Clade, pestivirus

## Abstract

Atypical porcine pestivirus (APPV), currently classified as *pestivirus K*, causes congenital tremor (CT) type A-II in piglets. Eighteen APPV strains were identified from 2297 South Korean wild boars captured in 2019. Phylogenetic analysis of the structural protein E2 and nonstructural proteins NS3 and Npro classified the APPV viruses, including reference strains, into Clades I, II and III. Clade I was divided into four subclades; however, the strains belonging to the four subclades differed slightly, depending on the tree analysis, the NS3, E2, and Npro genes. The maximum-likelihood method was assigned to South Korean wild boar APPV strains to various subclades within the three trees: subclades I.1 and I.2 in the E2 tree, subclade I.1 in the Npro tree, and subclades I.1 and I.4 in the NS3 ML tree. In conclusion, APPV among South Korean wild boars belonging to Clade I may be circulating at a higher level than among the South Korean domestic pig populations.

## 1. Introduction

Pestiviruses are highly variable single-stranded RNA genome viruses belonging to the family *Flaviviridae*. The genus *Pestivirus* includes animal pathogens that are of worldwide socioeconomic significance—these include bovine viral diarrhea virus (BVDV, *pestivirus A–B*), classical swine fever virus (CSFV, *pestivirus C*), and border disease virus (BDV, *pestivirus D*) [1]. Other *Pestiviruses* include *Pestivirus E* (pronghorn pestivirus), *Pestivirus F* (Bungowannah virus), *Pestivirus G* (giraffe pestivirus), *Pestivirus H* (Hobi-like pestivirus), *Pestivirus I* (Aydin-like pestivirus), and *Pestivirus J* (rat pestivirus) [1].

A novel genetically distinct strain of *pestivirus K*, named atypical porcine pestivirus (APPV), was first identified in the USA in 2015 [2]. It was also identified in Germany, the Netherlands, Austria, Spain, China, Hungary, Brazil, and Sweden in 2016–18 [3,4,5,6,7,8]. In South Korea, the first case of APPV, which causes congenital tremor (CT) type A-II in suckling piglets, was identified in 2017 [9]. Although APPV has been found in animals with no clinical signs, there is clear evidence that it is associated with CT type A-II in newborns [10]. The presence of APPV in domestic pigs was confirmed repeatedly in several countries in Europe, North and South America, and Asia [2,11], and in wild boars from countries in Europe [10,12,13]. Clinically, healthy pigs and wild boars may have an epidemiological role as vehicles for APPV.

*Pestivirus* genomes can be classified into genogroups and sub-genogroups [14,15]. Phylogenetic analysis of the E2 and Npro proteins divided APPV into at least four (A–D) [5,16] or five (A–E) different genogroups [17]. 

In this study, we describe the detection and genetic characterization of APPV in wild boars from South Korea.

## 2. Material and Methods

### 2.1. Wild Boars

To satisfy the OIE requirements for the surveillance of wild boars and feral pigs in Classical Swine Fever-free countries, wild boars have been hunted (in co-operation with the Korean Pork Producers Association and the South Korea government) since 2010. Immediately after hunting, blood samples were collected from wild boars captured in nine provinces of South Korea and transported to the APQA. Blood samples from 2297 wild boars (1126 males, 1045 females, and 126 unknown), captured in 2019, were screened for APPV.

### 2.2. Reverse Transcription-Polymerase Chain Reaction (RT-PCR) of APPV

RT-PCR was performed to detect APPV [12,16]. Briefly, total RNA was extracted from 100 μL of whole blood using the QIAamp viral RNA mini kit (Qiagen, Cat. No. 52904. Hilden, Germany). Extracted RNA was reverse-transcribed using SuperScript III (Invitrogen, Cat. No. 18080093. Carlsbad, CA, USA). For APPV screening and sequencing, PCR was performed using primers designed to target areas of the conserved NS3-encoding region, as described previously [12]. Primers targeting the E2 and Npro genes were used to amplify the complete nucleotide sequences, as described previously [16]. The amplification products were purified using the QIAquick Gel Extraction Kit (Qiagen, Cat. No. 28704. Hilden, Germany) and used directly for sequencing (Cosmogentech Co., Seoul, Korea). PCR and serum neutralization tests were used to detect viral antigens and antibodies specific for CSFV and BVDV in APPV-positive samples, as described previously [18].

### 2.3. Phylogenetic Analysis of APPV

Multiple nucleotide sequence alignment was carried out by the Clustal X alignment program [19] using APPV sequences available in GenBank as references, and BLAST software (NCBI, Bethesda, Rockville, MD, USA). Outgroup strains comprised *pestiviruses A–H*. The partial sequences of NS3, derived from 18 APPVs detected in South Korean wild boars, were compared with 86 reference sequences (including eight outgroup strains) from Asia, North America, and Europe. The complete E2 sequence of four APPVs and the complete Npro sequence data of five APPVs detected in South Korean wild boars were compared with 70 (including ten outgroup strains) and 69 reference sequences (including ten outgroup strains), respectively. Nucleotide sequences of the NS3, E2, and Npro regions were analyzed phylogenetically using the maximum-likelihood (ML) method, with the Tamura–Nei model and bootstrap analysis (*n* = 1000) within MEGA 7.0 software (State College, PA, USA) with default parameters [20]. The ML tree was based on rates among sites (Gamma distributed with invariant sites (G+I)) and the ML heuristic method (Nearest-neighbor-interchange (NNI)). The partial NS3 sequences of 18 APPV strains (accession numbers: MT501737–MT501754), the complete E2 sequences of four APPV strains (accession numbers: MT501733–MT501736), and the complete Npro sequences of five APPV strains (accession numbers: MT501555–MT501759) detected in South Korean wild boars were deposited in GenBank.

### 2.4. Ethical Approval

The authors confirm that the work complies with the ethical policies of the journal. The work was approved by the Institutional Animal Care and Use Committee of the Animal and Plant Quarantine Agency (APQA) (Approval Number: 2019-448).

## 3. Results 

### 3.1. Geographic Prevalence of APPV

**Figure 1:**
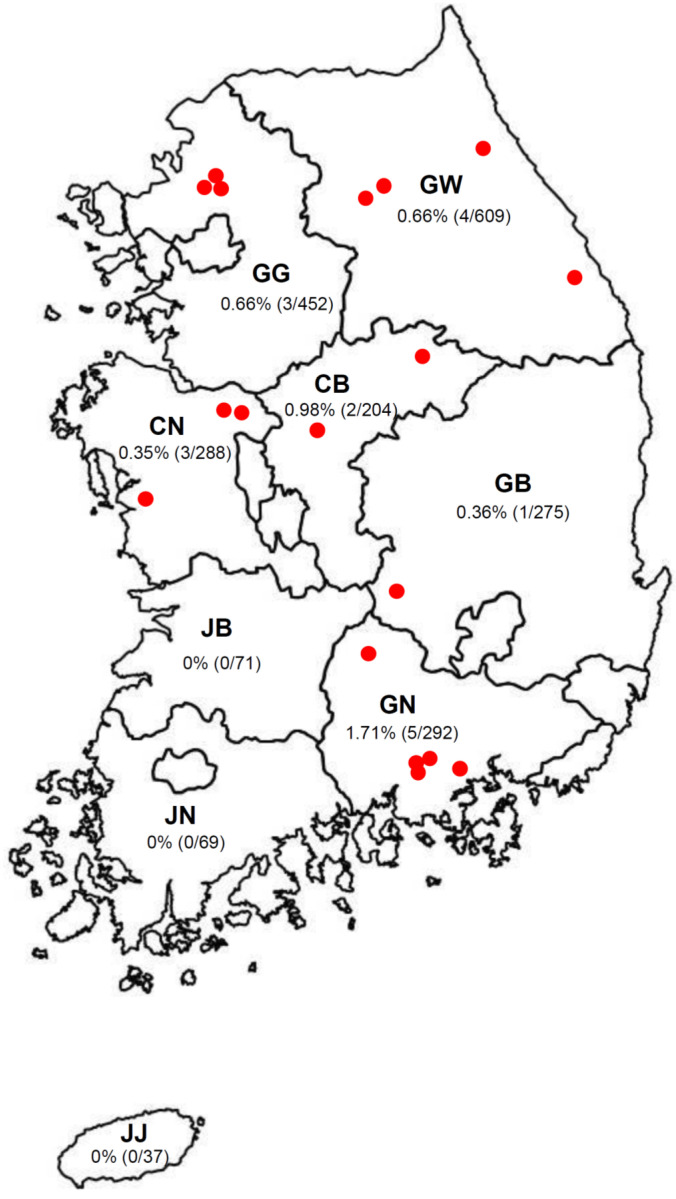
Locations in which APPV-positive wild boars were captured. Locations in which wild boars were captured are marked by a red dot. GW: Gangwon; GG: Gyeonggi; GN: Gyeongnam; GB, Gyeongbuk; JN: Jennam; JB: Jenbuk; CN: Chungnam; CB: Chungbuk; JJ: Jeju.

Eighteen APPV strains were identified in 2297 blood samples collected from wild boars in 2019, suggesting that the prevalence of APPV is 0.78%. Of the APPV-positive wild boars, 15 were male (15/18, 83.3%), two were female (2/18, 11.1%), and one was of unknown sex (1/18, 5.6%). APPV strains were detected in wild boars from six provinces and of various weights (seven <30 kg; seven 30–60 kg; four >60 kg). Among the 18 APPVs detected in South Korean wild boars, five were detected in Gyeongnam (GN, 5/292; 1.71%), four in Gangwon (GW, 4/609; 0.66%), three each in Gyeonggi (GG, 3/452; 0.66%) and Chungnam (CN, 3/288; 0.35%), two in Chungbuk (CB, 2/204; 0.98%), and one in Gyeongkuk (GB, 1/275; 0.36%), as shown in Figure 1. All APPV-positive samples were negative for anti-CSFV and BVDV antibodies and antigens.

### 3.2. ML Trees Based on NS3 Sequences

**Figure 2:**
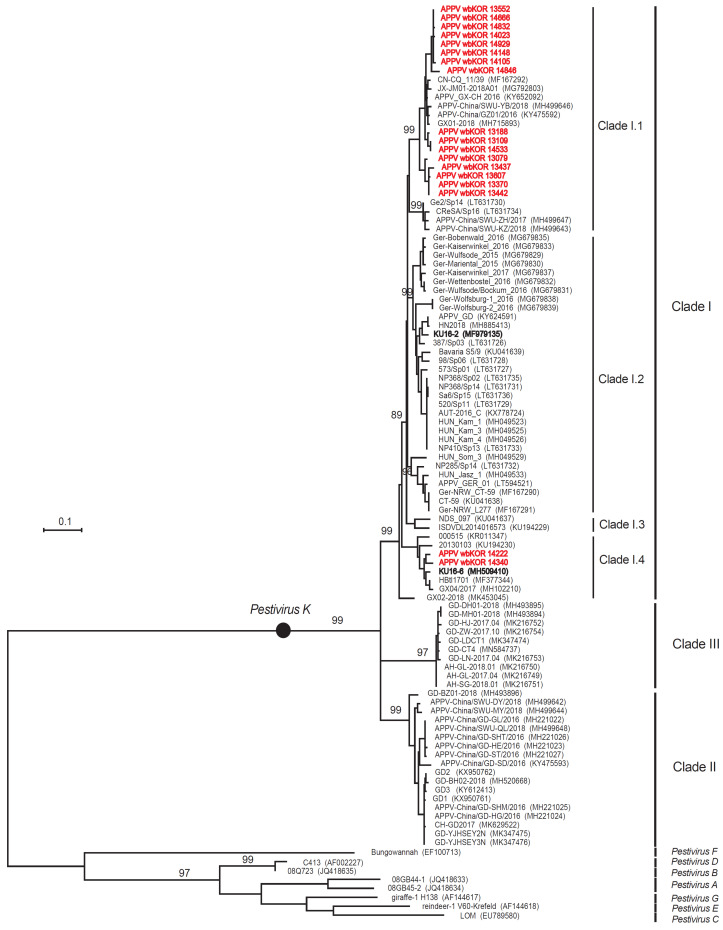
Phylogenetic tree of South Korean wild boar APPVs, based on NS3 sequences. The phylogenetic tree was constructed using the ML method (based on the Tamura–Nei model), with bootstrap analysis (*n* = 1000), in MEGA 7.0 software. The 767 nt NS3 sequences of 18 APPVs from South Korean wild boars were compared with 86 reference sequences (including eight outgroup strains: *pestiviruses A–G*) from Asia, North America, and Europe. The Log likelihood (Log L) is −10,191.77, and only bootstrap values ≥70 are indicated on the nodes. South Korean wild boar APPV strains and South Korean domestic pig APPV strains are denoted by red bold and black bold letters. The scale bar indicates the number of nucleotide substitutions per site.

**Figure 3:**
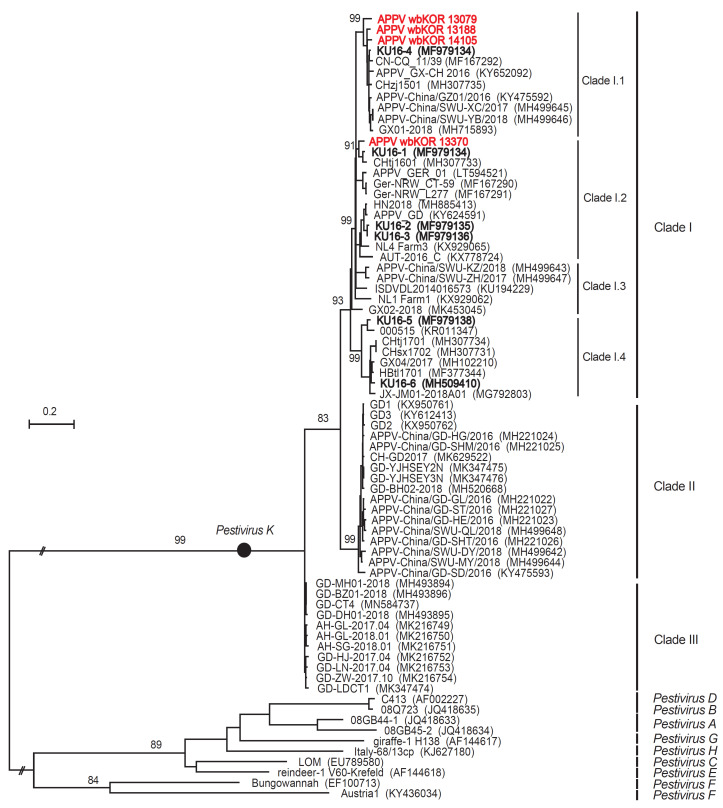
ML tree of South Korean wild boar APPVs, based on E2 sequences. The phylogenetic tree (Log L, −11,438.12) was constructed using the ML method (based on the Tamura–Nei model), with bootstrap analysis (*n* = 1000). The complete E2 sequences of four APPVs detected in South Korean wild boars were compared with 70 reference sequences (including ten outgroup strains: *pestiviruses A–H*).

**Figure 4:**
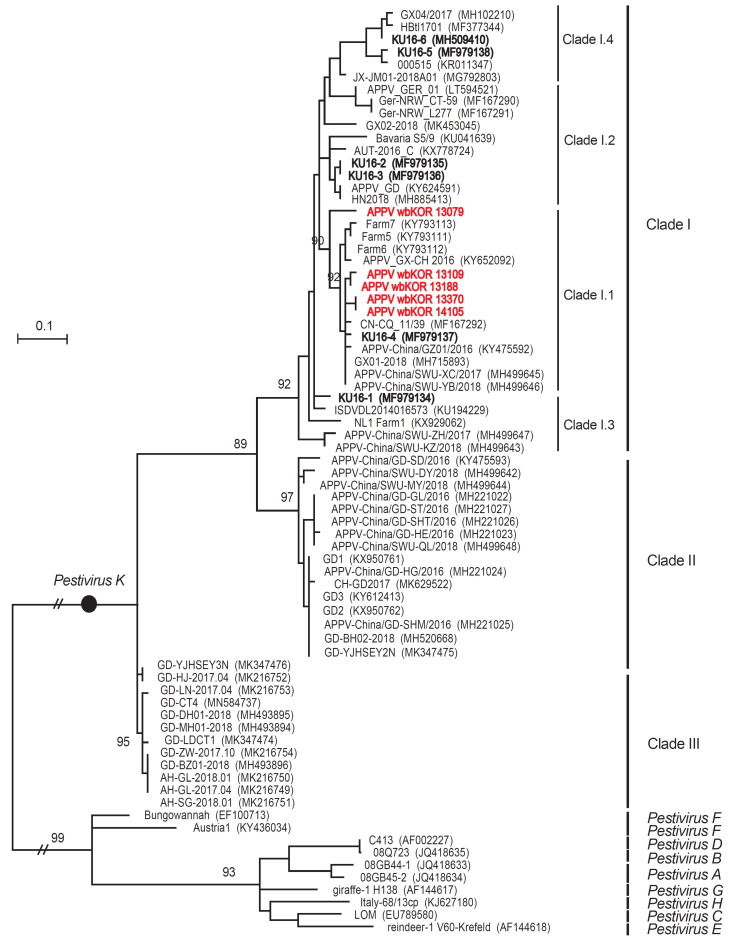
ML tree of South Korean wild boar APPV, based on Npro sequences. The phylogenetic tree (Log L: −5113.82) was constructed using the ML method (based on the Tamura–Nei model), with bootstrap analysis (*n* = 1000). The complete Npro sequences of five APPV detected in South Korean wild boars were compared with 69 reference sequences (including ten outgroup strains: *pestiviruses A–H*).

The NS3 sequences (767 nucleotides (nt)) of the 18 APPVs detected from wild boars were 87.7–99.9% identical at the nt level and 96.5–100% identical at the amino acid (aa) level. ML tree analysis of NS3, E2, and Npro sequences revealed that *Pestivirus* strains were clearly divided into two groups: *Pestivirus K* (APPVs) and Other *Pestiviruses* (*A*–*H*), as shown in Figure 2, Figure 3 and Figure 4). All APPVs were classified into three large Clades (I, II, and III) and four smaller subclades (I.1, I.2, I.3, and I.4), as shown in Figure 2, Figure 3 and Figure 4). ML analysis of the NS3 sequences of the 18 APPVs from South Korean wild boars were included in Clade I (16 in subclade I.1 and two in subclade I.4), as shown in Figure 2. The nt sequence identity between the South Korean APPVs in subclades I.1 and I.4 was 86.8–88.7%; however, identity at the aa sequence level was 95.7–97.6%.

The 767-nt NS3 sequences of the 16 South Korean wild boar APPVs (Subclade I.1) and 8 APPVs isolated from German wild boars (Subclade I.2), as shown in Figure 2, were 90.9–92.0% identical at the nt level and 97.6–99.2% identical at the aa level. Moreover, NS3 sequences from the two South Korean wild boar APPVs (subclade I.4) and of the eight German wild boar APPVs (subclade I.2) were 88.1–88.9% identical at the nt level and 96.1–97.3% identical at the aa level.

### 3.3. ML Trees for the E2 Sequences

The E2 sequences (723 nt) from four South Korean wild boar APPVs (wbKOR 13079, -13188, -14105, and -13370) were 91.3–94.3% identical at the nt level and 95.4–97.1% identical at the aa level. ML analysis of the E2 sequences categorized them as Clade I (three in subclade I.1 and one in subclade I.2), as shown in Figure 3. By contrast, six APPVs from South Korean domestic pigs belonged to three subclades (one to subclade I.1, three to subclade I.2, and two to subclade I.4), as shown in Figure 3. The four South Korean wild boar APPVs and six South Korean domestic pig APPVs were 87.4–94.3% identical at the nt level and 94.2–98.8% identical at the aa level. The E2 sequences from the four South Korean wild boar APPVs and the 17 other APPVs in Clade II were 84.4–85.1% identical at the nt level and 94.2–96.3% identical at the aa level, whereas the four South Korean wild boar APPVs and 11 other APPVs in Clade III were 81.5–82.7% identical at the nt level and 90.0–92.5% identical at the aa level.

### 3.4. ML Trees for the Npro Sequences

The Npro sequences (540 nt) of five APPVs from South Korean wild boars (wbKOR 13079, -13109, -13188, -14105, and -13370) were 92.6–98.7% identical at the nt level and 93.9–99.4% identical at the aa level. ML analysis of the Npro sequences categorized them as Clade I (all five belonged to subclade I.1), as shown in Figure 4. The six APPVs from South Korean domestic pigs were distributed among all subclades (one in subclade I.1, two in subclade I.2, one in subclade I.3, and one in subclade I.4), as shown in Figure 4. The nt sequence identity between the five South Korean wild boar APPVs and the six South Korean domestic pig APPVs was 85.0–95.4%, whereas the aa sequences were 89.4–95.0% identical. The Npro sequences of the five South Korean wild boar APPVs and the 16 APPVs in Clade II were 79.3–80.5% identical at the nt level and 80.6–83.9% identical at the aa level, whereas the sequences from the five South Korean wild boar APPVs and the 12 APPVs in Clade III were 77.0–80.4% identical at the nt level and 80.6–81.1% identical at the aa level.

## 4. Discussion

Some retrospective studies suggest that APPVs were circulating widely for decades before the recent reports describing their detection [21,22]. An early study identified an APPV strain from samples obtained from piglets with CT; qRT-PCR of samples from a pig herd in the USA revealed an APPV prevalence of 6% [2]. Another study reported APPVs in CT-affected piglets from many countries, which further supports the potential relationship between APPV and CT type A-II [3]. In South Korea, APPV sequences from the domestic pig samples collected in 2016 had been submitted to GenBank (accession numbers: MF979135, MH509410), and the first identification of APPV was reported in suckling piglets with CT in 2017 [9]. Moreover, an APPV detection rate of 2.4–22% was reported in apparently healthy pigs in the USA and Germany [6,12,23]. A recent study of serum samples from apparently healthy pigs revealed that the prevalence of APPV in Europe was 2.3–17.5%, and that in China it was 5–11% [11]. All of these results demonstrate that APPV is highly prevalent in both apparently healthy pigs and CT-affected pigs, suggesting that the virus may have spread worldwide. 

Wild boars are susceptible to APPV infection, although the role of this species in the epidemiology of the virus is unknown [13]. A very recent finding is that the prevalence of APPV in serum samples from wild boars in Europe is 0.23–19%, suggesting that wild boars may be a reservoir for APPV [10,12,13]. All wild boars sampled in this study, including APPV-positive wild boars, were considered healthy (based on their behavior) before hunting. The prevalence of APPV among South Korean wild boars is low, as in Spain (0.23%) and Italy (0.69%) [10,13]; however, this is in contrast to the high prevalence detected in northern Germany (19%) [12]. Furthermore, serological investigations in wild boars revealed an antibody detection rate of 52% in northern Germany, where APPV seems to be endemic among wild boars in many areas [12].

Phylogenetic analysis revealed that APPV sequences (complete or partial polyprotein) exhibit high genetic diversity between strains detected in different countries and species (domestic pigs or wild boars), and that they form independent clusters according to geographic location [24]. 

Recently, phylogenetic analysis in China revealed a high level of genetic variation among APPVs: three Clades (I–III), with four subgroups (1–4) in Clade I [25]. Sequence analysis of the APPV NS3, E2, and Npro genes, followed by the construction of ML trees, classified them into three Clades—APPVs isolated from China were assigned to all Clades, whereas APPVs isolated from Asia, North America, and European countries belonged only to Clade I [25,26]. APPV strains isolated from South Korean wild boars and South Korean domestic pigs belonged to Clade I, without a preference for any particular subclade. This means that APPVs may have high the potential for spreading between wild boars and domestic pigs. In the three ML trees (NS3, E2, and Npro) constructed for this study, Clade I contained four subclades; however, the APPV strains contained within these subclades were not clearly distinguished by the three ML trees. A previous study suggests that recombination events occur between Clades (Clades II and III) or within a Clade (Clade I) [25]. Therefore, further research is needed to determine whether the difference in subclade of South Korean APPV strains in three ML trees are because of the recombination events within Clade I.

## 5. Conclusions

Here, we present the first report of APPV detected in South Korean wild boars. We found that the overall prevalence of APPV in South Korean wild boars was low (0.78%). South Korean wild boars harbor genetically diverse APPV strains belonging to Clade I. Wild boars may be an important virus reservoir for APPV. More epidemiological information will help to establish effective control measures and to eradicate the virus from affected pig herds in the future.
